# Identifying cancer cell‐secreted proteins that activate cancer‐associated fibroblasts as prognostic factors for patients with pancreatic cancer

**DOI:** 10.1111/jcmm.17596

**Published:** 2022-10-25

**Authors:** Qiankun Luo, Jiayin Liu, Qiang Fu, Xu Zhang, Pengfei Yu, Pan Liu, Jiali Zhang, Huiyuan Tian, Song Chen, Hongwei Zhang, Tao Qin

**Affiliations:** ^1^ Department of Hepatobilliary and Pancreatic surgery Zhengzhou University People's Hospital, Henan Provincial People's Hospital Zhengzhou China; ^2^ Academy of Medical Sciences, Zhengzhou University Zhengzhou China; ^3^ Department of Research and Discipline Development Henan Provincial People's Hospital, Zhengzhou University People's Hospital Zhengzhou China; ^4^ Translational Research Institute, Henan Provincial People's Hospital, Zhengzhou University People's Hospital, and Molecular Pathology Center Academy of Medical Sciences, Zhengzhou University Zhengzhou China; ^5^ Henan University People's Hospital Zhengzhou China

**Keywords:** cancer‐associated fibroblast, pancreatic cancer, pancreatic stellate cells, prognosis, proteins

## Abstract

The study aimed to investigate the mechanism by which cancer‐associated fibroblasts (CAFs) are activated by cancer cells and construct a risk model to predict the prognosis of patients with pancreatic cancer (PC) after surgery. Pancreatic stellate cells were isolated from human pancreatic tissue and co‐cultured with cancer cells to verify their crosstalk. Liquid chromatography–tandem mass spectrometry was used to detect proteins secreted by cancer cells. The online tools Gene Expression Profiling Interactive Analysis, UALCAN, and the Human Protein Atlas were used to analyse gene expression in PC. Expression data from the cancer genome atlas and the clinical samples were used to develop a training receiver operating characteristic (ROC) model and an external validation ROC model, respectively. We identified that cancer cells promote the activation of inflammatory CAFs (iCAF) through secretory proteins, which promote PC metastasis. Six candidate proteins secreted by cancer cells were identified which promote iCAF formation. These proteins were highly expressed in tumours and were associated with a poor prognosis in patients with PC. Moreover, a 6‐gene model was constructed to predict death risk in patients at 1, 2 and 3 years after surgery. The training areas under the ROC curves (AUC) of 1‐, 2‐ and 3‐year death risks were 0.780, 0.792 and 0. 825, respectively. The externally validated AUC of death at 3 years post‐surgery was 0.728. In conclusion, cancer cell‐secreted proteins play a vital role in iCAF formation, and the 6‐gene model may be a potential marker for predicting whether PC patients will benefit from surgery.

## 1 INTRODUCTION

Pancreatic cancer (PC) is a highly aggressive disease. The 5‐year survival rate is <9% for all stages and is the lowest among all cancers according to the data of Cancer Statistics 2019.^1^ Surgical resection is the only potential curative treatment for PC.^2^ However, more than 80% of patients who underwent surgical resection develop metastasis and recurrence shortly after surgery.^3^ Therefore, there is a pressing need to identify novel markers to predict prognosis and provide guidance for patients who will benefit from surgical treatment.

Cancer‐associated fibroblasts (CAFs) in PC, generally believed to originate from pancreatic stellate cells (PSCs), are composed of various subtypes and play vital roles in cancer progression.^4^ Recent studies have demonstrated that some CAFs promote the growth, invasion and chemoresistance of PC, whereas other populations can restrain tumour activity.^4^, ^5^, ^6^ Although alpha‐smooth muscle actin (α‐SMA) and platelet‐derived growth factor receptor A (PDGFR‐α) have been reported to be specific markers for CAFs, specific markers to determine CAF subtypes that play a malignant role in PC progression remain to be defined.^7^, ^8^ Moreover, inflammatory CAFs (iCAF) with a high expression of interleukin (IL)‐6 were identified to promote tumour progression.^9^, ^10^ Inflammatory markers, including IL‐11 and IL‐1α, and leukaemia inhibitory factor (LIF) are considered to be markers of iCAF.^9^ Furthermore, α‐SMA+ stromal cells secrete IL‐6, inducing liver metastasis via the activation of signal transducer and activator of transcription 3 (STAT3).^11^ Moreover, blockade of IL‐6 signalling can inhibit tumour activity and enhance the antitumor activity of PD‐L1 antibodies in murine models of PC.^11^, ^12^ Therefore, iCAF may play a critical role in PC metastasis and malignant behaviours.

Many researchers have focused on the crosstalk between PC cells and CAFs.^6^, ^13^ However, most studies have reported that CAFs contributed to the malignant activity of tumours through secretory proteins or direct interactions.^4^, ^14^, ^15^ The mechanisms by which cancer cells promote CAFs formation have not been completely clarified. Here, we investigated the cancer cell secretory proteins that may induce CAF activation and determined the role of iCAF in the promotion of PC invasion. We also presented a 6‐gene model to predict PC prognosis. These findings provide a novel therapeutic strategy targeting the PC tumour stroma or prognostic markers for the surgical treatment of patients with PC.

## 2 MATERIALS AND METHODS

### 2.1 Cell culture and the isolation of PSCs

The human PC cell line MIApaca‐2, BxPC‐3, SW1990 and PANC‐1 cells were purchased from the National Collection of Authenticated Cell Cultures (Shanghai). PSCs were isolated from pancreatic tissues adjacent to PC tumours using the outgrowth method and immortalized as reported by Tuveson et al^10^ Cells were cultured as previously described.^16^ All patients signed informed consent.

### 2.2 Transwell co‐culture assay

Cancer cells were plated in the top chamber at 1 × 10^4^ per well in serum‐free medium. In the bottom chamber, 5 × 10^5^ cancer cells or PSCs were seeded in media containing 10% foetal bovine serum (FBS). After co‐cultured for 24 h, the cells in the top chamber were fixed with 4% paraformaldehyde for 15 min and stained with 0.5% crystal violet for 15 min. ImageJ was used for the cell count.

### 2.3 Condition‐media (CM)

When the density of cancer cells reached 80%, the cells were washed, and serum‐free media were added. After 24 h, the supernatant was collected as CM and stored in – 80°C for proteomic analysis.

### 2.4 Immunocytochemistry (ICC) and immunofluorescence (IF) staining

For ICC, cells were seeded on a glass coverslip and cultured with control medium or CM from MIApaca‐2, BxPC‐3, SW1990 or PANC‐1 for 24 h. Cells were washed and fixed. Triton‐X‐100 was used to permeabilize the cells. After treating with 3% hydrogen peroxide, cells were incubated with antibodies against α‐SMA (ab32575, 1:100) and PDGFR‐α (ab203491, 1:100) from Abcam overnight, and horseradish peroxidase‐linked secondary antibodies for 1 h. Diaminobenzidine (DAB) was used for detection. For IF, cells were treated as in ICC and incubated with antibodies against α‐SMA (ab32575, 1:100), E‐cadherin (ab219332, 1:100), fibroblast activation protein (FAP, ab53066, 1:100) and epithelial cell adhesion molecule (EpCAM) (ab218448, 1:100) from Abcam overnight. The cells were then incubated with the secondary antibodies, Alexa Fluor 488 (A32731, Invitrogen) and Alexa Fluor 594 (A48271, Invitrogen). Subsequently, the coverslips were fixed and imaged.

### 2.5 Oil Red‐O staining

PSCs were seeded on a glass coverslip for 3 days and fixed as previously reported.^10^ After washing, the cells and nuclei were stained using Oil Red‐O working solution and haematoxylin following the instructions (C0157S, Beyotime).

### 2.6 Western blot (WB)

When the cell density of PSCs reached 80%, the medium was replaced with Dulbecco's modified eagle medium (DMEM) containing 5% foetal bovine serum (FBS) in the control group and 50% DMEM containing 10% FBS added with 50% CM from MIApaca‐2, SW1990 or PANC‐1 in the CM group for 24 h. Total protein was extracted and analysed using WB as previously described.^16^ The antibodies used for WB were PDGFR‐α (ab203491, 1:500), FAP (ab53066, 1:100) and α‐SMA (ab32575, 1:500) from Abcam.

### 2.7 Contraction assay

Rat‐tail tendon collagen (5 mg/ml; Solarbio) was diluted to 2.5 mg/ml. PSCs (1 × 10^5^) were seeded into 1 ml of rat‐tail tendon collagen in a 96‐well plate.^17^ After polymerization for 12 h, the collagen was cultured in DMEM containing 5% FBS in the control group, and in 50% DMEM containing 10% FBS with 50% CM from MIApaca‐2, SW1990 or PANC‐1 in the CM group. After 3 days, puromycin was used to remove CAFs from the matrix.

### 2.8 Enzyme‐linked immunosorbent assay (ELISA)

When the cell density of PSCs reached 80%, the medium was replaced with DMEM containing 5% FBS in the control group, and 50% DMEM containing 10% FBS with 50% CM from MIApaca‐2, SW1990 or PANC‐1 in the CM group. After 48 h, the cell supernatant was collected and purified. The quantification of IL‐6, IL‐11 and LIF was performed using ELISA kits (IL‐6, VAL106, R&D systems, USA; IL‐11, D1100, R&D systems; LIF, E‐EL‐H0094c, Elabscience Biotechnology Co., Ltd). The level of IL‐6, IL‐11 and LIF was subtracted from CM co‐cultured with CAFs to CM generated by cancer cells.

### 2.9 Invasion assay

Two days after contraction, the matrix was moved to the top chamber of the transwell. 1 × 10^4^ cancer cells were seeded on the matrix. DMEM containing 10% FBS was added into the bottom chamber. The cancer cells were cultured for 10 days. The matrix was stained with haematoxylin and eosin (H&E).

### 2.10 Liquid chromatography–tandem mass spectrometry (LC–MS/MS)

The CM from MIApaca‐2, SW1990, PANC‐1 cells were centrifuged to remove cellular debris. The protein sample was reduced with dithiothreitol, alkylated with iodoacetamide and diluted with triethylammonium bicarbonate (TEAB) to a urea concentration of <2 M. The proteins were digested overnight with trypsin. The peptides were desalted using C18 SPE column, reconstituted in TEAB and labelled with a 0.5 M tandem mass tag (TMT) reagent (Thermo Fisher). After incubation for 2 h, the samples were quenched, desalted using C18 SPE column and dried by vacuum centrifugation. Finally, the peptides were analysed by LC–MS/MS using an EASY nLC 1200 and Orbitrap Exploris™ 480 (Thermo Fisher).^18^ The differentially expressed proteins between MIApaca‐2, SW1990 and PANC‐1 were defined as fold change > 1.3 and *p* < 0.05. Gene ontology (GO) enrichment was analysed using UniProt‐GOA database (http://www.ebi.ac.uk/GOA/).

### 2.11 Quantitative real‐time PCR (RT‐qPCR) of clinical samples and bioinformatics analysis

**FIGURE 1:**
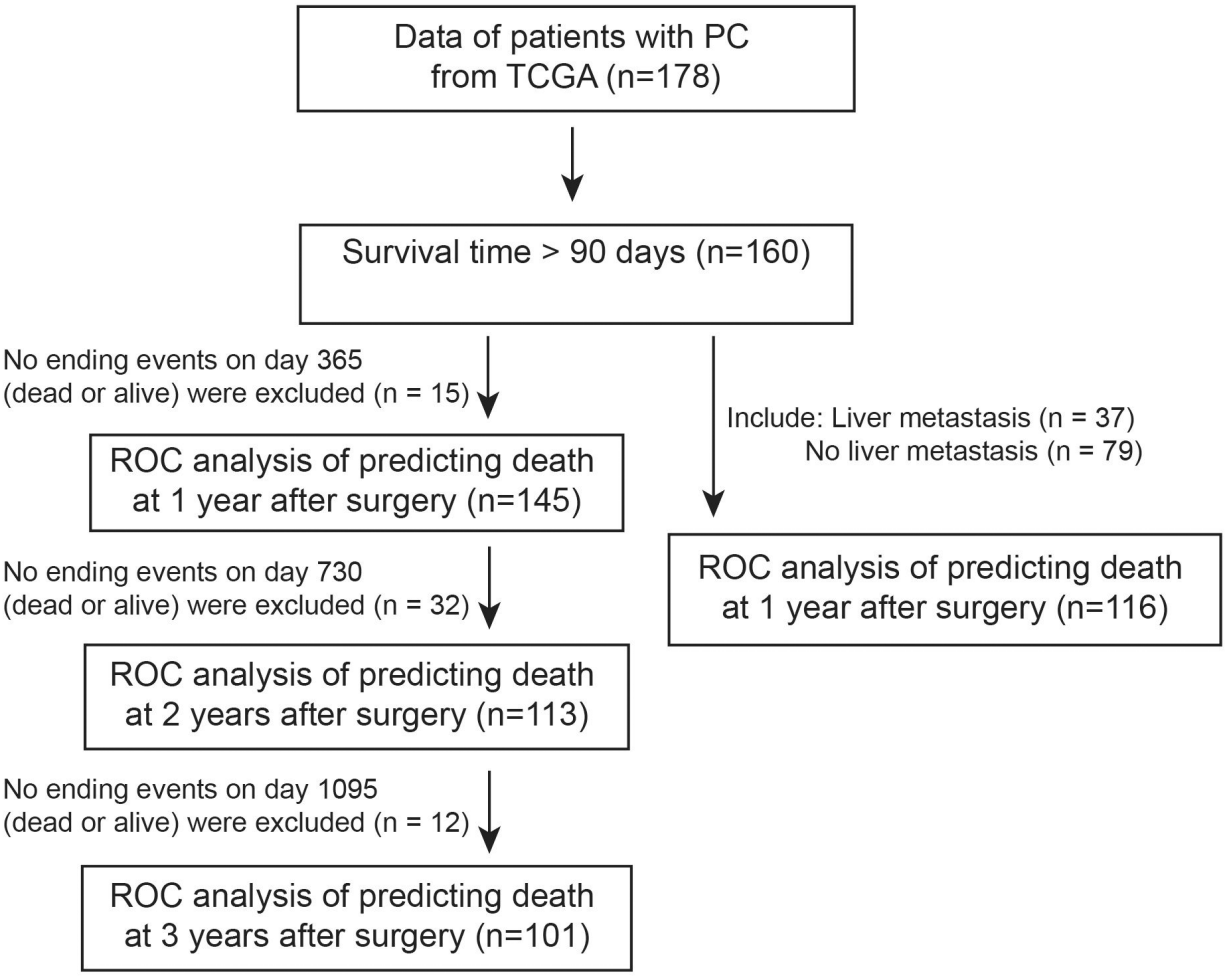
Workflow of the training cohort for the receiver operating characteristic (ROC) analysis

The tumour tissues of 52 PC patients with PC from Henan Provincial People's Hospital were collected as previously described.^16^ Total RNA was extracted and reverse‐transcribed as previously reported.^16^ RT‐qPCR was performed using SYBR qPCR Master Mix (Vazyme). The primers were provided in the Table S1. Survival related genes were obtained from the cancer genome atlas (TCGA) database (http://www.cbioportal.org/index.do). Gene Expression Profiling Interactive Analysis (GEPIA) and UALAN were used for bioinformatics analysis.^19^, ^20^ The data of patients with PC from TCGA were used for the training model of receiver operating characteristic (ROC) analysis. Data were excluded or included as shown in the workflow (Figure 1).

### 2.12 Statistical analysis

Data were analysed using GraphPad Prism 5.0 and IBM SPSS Statistics 22.0. The comparison between two groups was assessed by two‐tailed Student's *t*‐test. Mann–Whitney U test was used for non‐normally distributed analysis. The prediction of death risks was assessed using ROC and area under the curve (AUC) value with 95% confidence interval (CI). *p* < 0.05 was considered statistically significant.

## 3 RESULTS

### 3.1 Identification of purified PSCs isolated from pancreatic tissues

**FIGURE 2:**
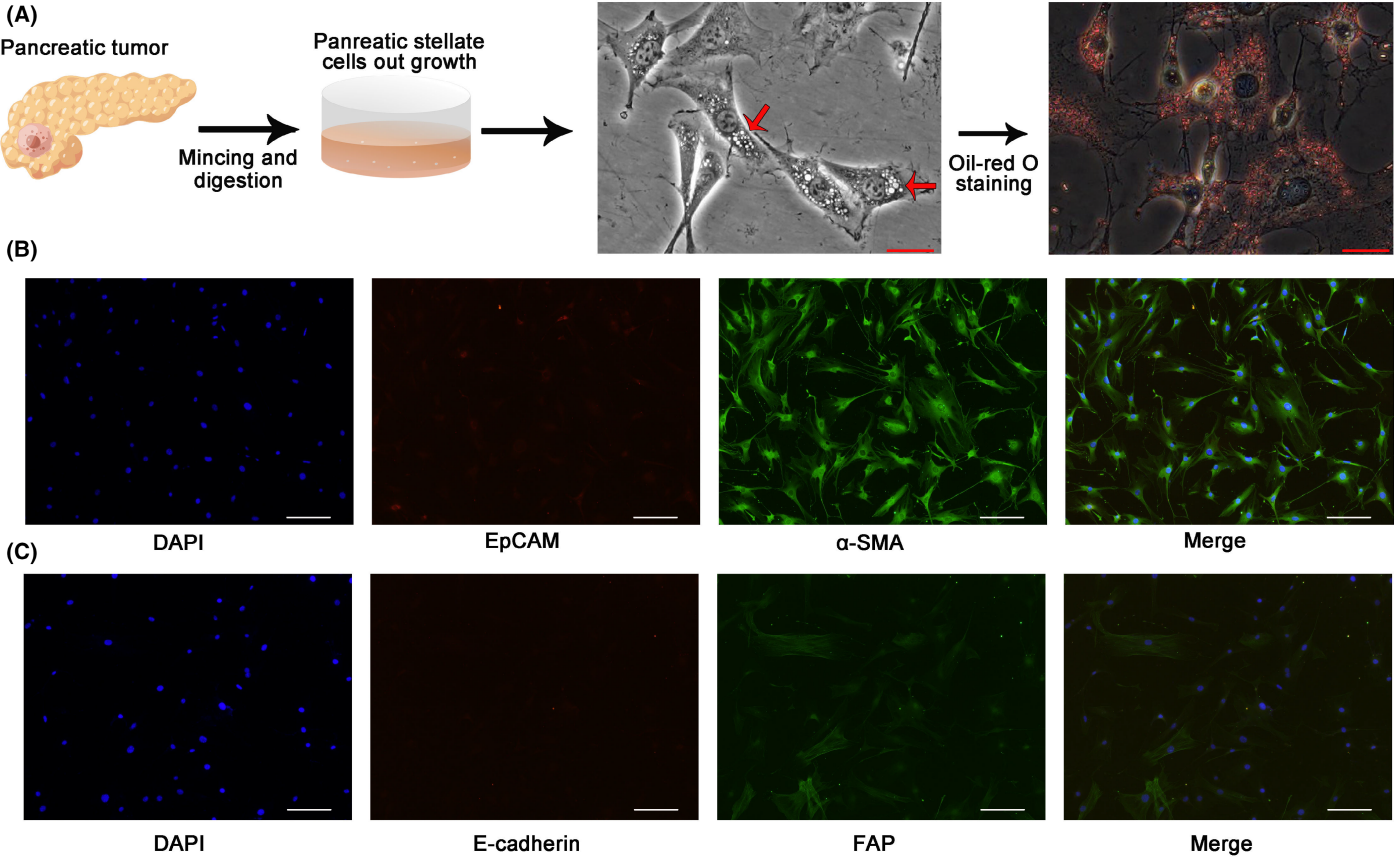
Isolation and identification of human pancreatic stellate cells (PSCs). (A) Schematic diagram of human PSCs isolation. The image of the pancreas was drawn by Figdraw (www.figdraw.com). The lipid droplets were observed under the microscope with or without oil‐red O staining. Bars, 50 μm. (B, C) Immunofluorescence staining of PSCs for fibroblast (α‐SMA, FAP) and epithelial markers (EpCAM, E‐cadherin). Bars, 50 μm

The role of PSCs was to store lipid droplets of pancreas. Thus, we performed Oil Red‐O staining to identify the PSCs according to the abundance of lipid drops. Many lipid drops were observed in the PSCs under a microscope. The presence of lipid drops was verified by Oil Red‐O staining (Figure 2A). IF results showed that PSCs were negative for EpCAM and E‐cadherin and were positive for α‐SMA and FAP (Figure 2B and C). These results confirmed that the cells isolated from pancreatic tissues were purified PSCs.^21^

### 3.2 The crosstalk between PSCs and cancer cells

**FIGURE 3:**
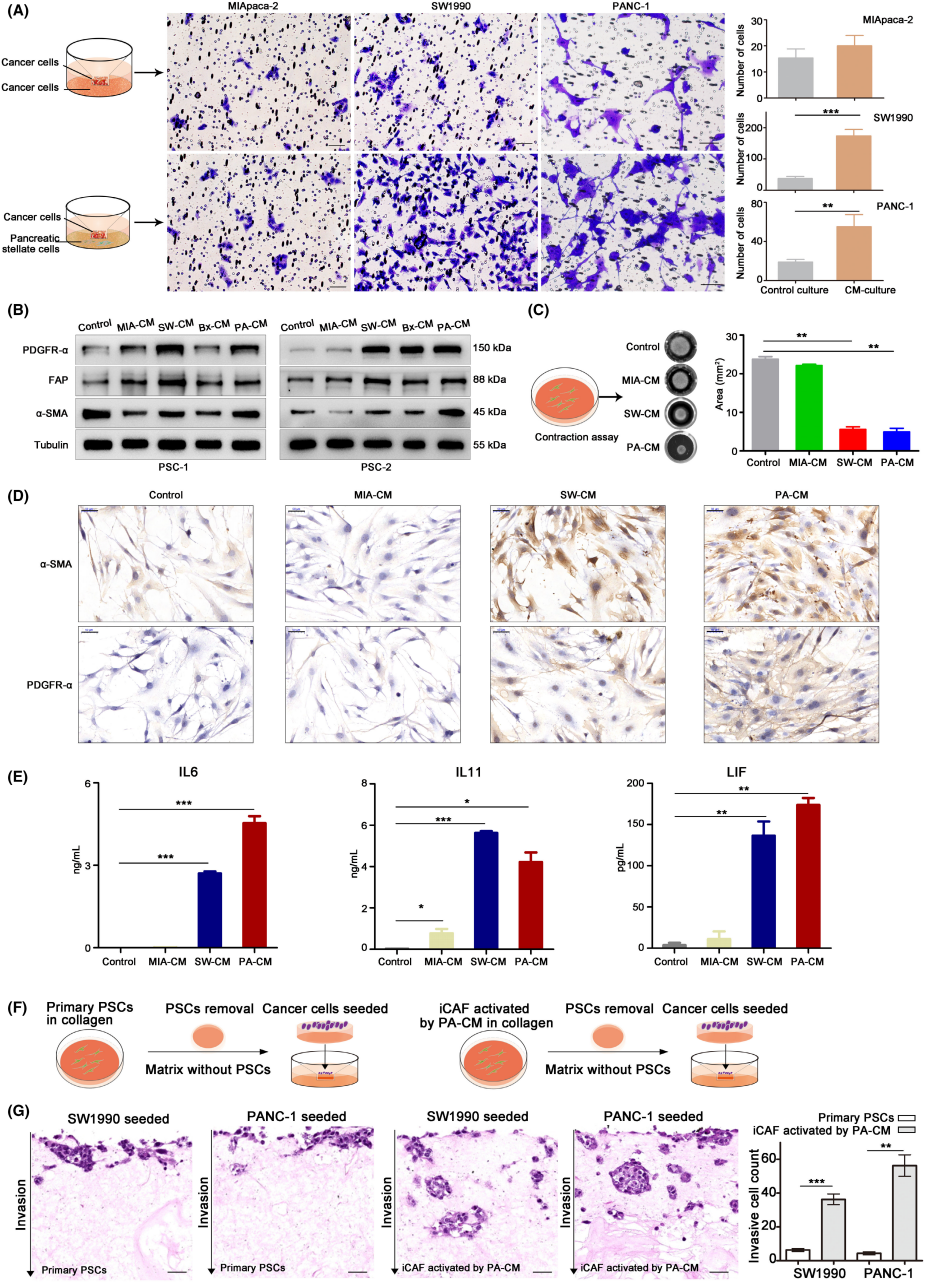
Crosstalk between cancer cells and PSCs. (A) Compared with cancer cells co‐cultured with cancer cells, SW1990 and PANC‐1 co‐cultured with PSCs showed high migration capacity. The number of migration cells increased significantly; however, there was no significant difference in MIApaca‐2. Bars, 50 μm. (B) Western blot analysis of PSCs (PSC1 and PSC2 were PSC separated from two patients) cultured with normal or condition media from MIApaca‐2 (MIA‐CM), SW1990 (SW‐CM), BxPC‐3 (Bx‐CM) and PANC‐1 (PA‐CM). Tubulin was used as a loading control. (C) Representative images of contraction collagen of PSCs incubated with normal media or MIA‐CM, SW‐CM and PA‐CM. The matrix area of three biological repeats was analysed. (D) Immunocytochemistry of PSCs cultured with normal media or MIA‐CM, SW‐CM and PA‐CM. Bars, 50 μm. (E) Enzyme‐linked immunosorbent assay of IL‐6, IL‐11 and LIF from the supernatant of PSCs treated with normal media or MIA‐CM, SW‐CM and PA‐CM. (F) Schematic diagram of PSCs‐based or iCAF‐based cancer cells invasion assay. The PSCs or activated iCAF were removed from matrix with puromycin and washed by PBS. (G) Representative haematoxylin and eosin staining of invasive cancer cells into matrix generated by PSCs or iCAF. Bars, 50 μm. The invasive cell count was analysed. All data analysis were performed using Student's *t*‐test. *p < 0.05, **p < 0.01, ***p < 0.0001

PSCs were co‐cultured with the PC cell lines MIApaca‐2, SW1990 and PANC‐1 in a Transwell to investigate the interaction between PSCs and PC cells. The results showed that the migration of SW1990 and PANC‐1 cells increased when co‐cultured with PSCs (*p* < 0.01, *p* < 0.001, respectively). However, the migration of MIApaca‐2 cells was not affected by the co‐culture (*p* = 0.47) (Figure 3A). Studies reported that PSCs could be activated to be CAFs that promoted cancer metastasis.^4^ Therefore, we supposed that SW1990 and PANC‐1 cells induced the formation of CAFs, but MIApaca‐2 cells did not. The hypothesis was confirmed to be true. The migration of MIApaca‐2 was improved by co‐culture with PSCs activated by CM from SW1990 (Figure S1).

Furthermore, we verified that pancreatic cancer cell secretory proteins activated PSCs into CAFs. PDGFRα is considered as a specific marker for CAFs. WB results indicated that the protein level of PDGFR‐α was upregulated after PSCs from two patients were treated with CM from SW1990 and PANC‐1 cells (Figure 3B). The activation effect of BxPC‐3 CM was unstable on CAFs. However, CM from MIApaca‐2 had very slight effect on CAF activation. ICC verified that the CM generated by SW1990 and PANC‐1 increased the expression of PDGFR‐α in CAFs (Figure 3D). The CAFs were especially activated by CM from SW1990 and PANC‐1. Contraction assay indicated that CM generated by SW1990 and PANC‐1 significantly enhanced the contractility of CAFs compared with CM generated by MIApaca‐2 and control media (Figure 3C).

Moreover, iCAF was activated by tumour‐secreted IL‐1 and then increased the secretion of IL‐6, IL‐11 and LIF^.^ ^9^ The expression of α‐SMA was down‐regulated in comparison with myofibroblastic CAFs (myCAF).^9^ ELISA was performed to confirm the presence of the iCAF subtype. The results showed that the activated CAFs secreted more IL‐6, IL‐11 and LIF. PSCs were activated to form iCAF (Figure 3 E). However, the CM from MIApaca‐2 cells did not promote iCAF formation. Subsequently, we verified that iCAFs promoted the invasion of PC cells via secreted factors. Cancer cells were seeded on the matrix generated by PSCs incubated with normal media or CM from PANC‐1 cells for invasion (Figure 3F). H&E staining demonstrated that the number of invasive cancer cells increased in the matrix treated with CM from PANC‐1 cells compared with normal media (Figure 3G), indicating that the matrix containing iCAF‐secreted factors promoted cancer cells invasion.

### 3.3 The identification of proteins secreted by cancer cells

**FIGURE 4:**
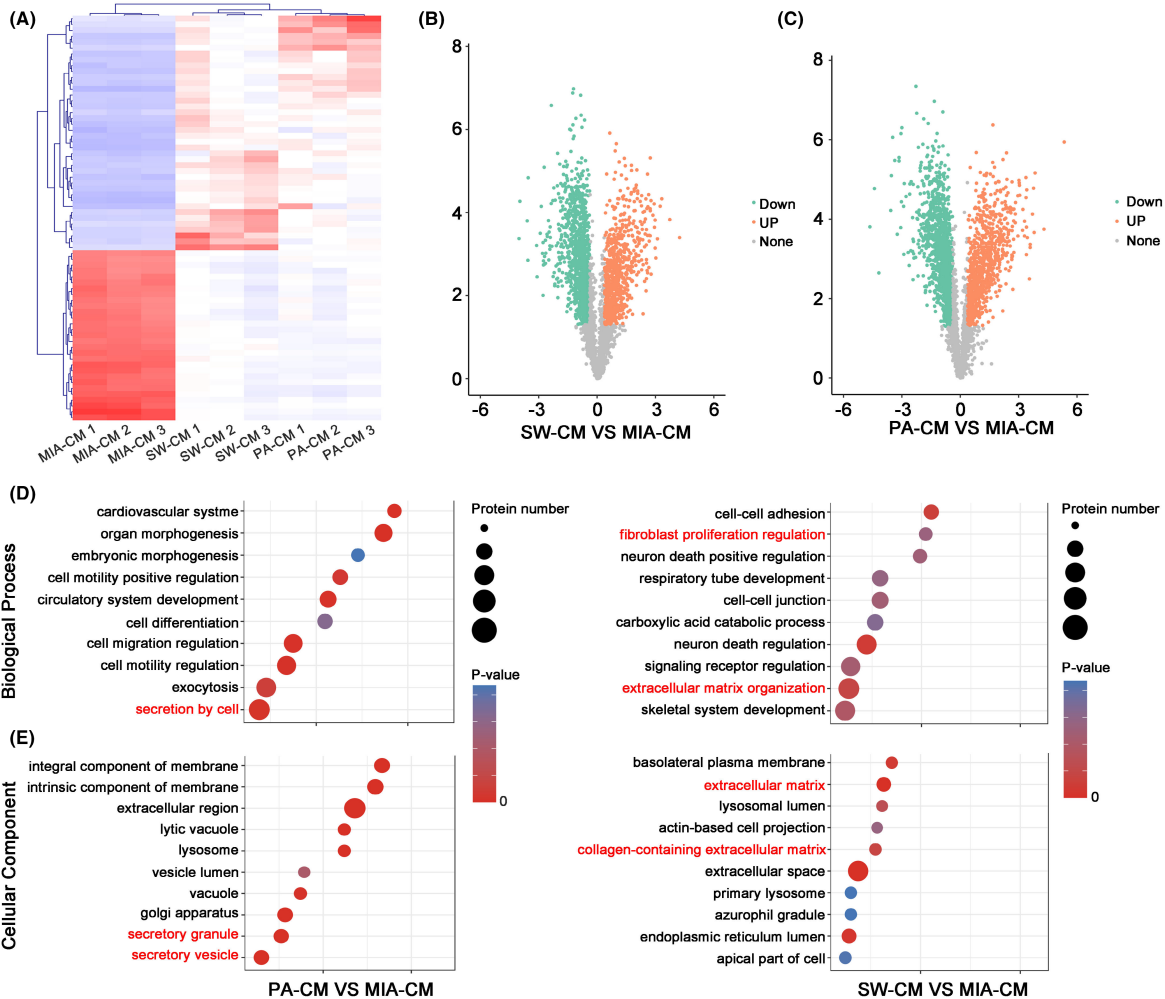
Analysis of liquid chromatography–tandem mass spectrometry (LC–MS/MS). (A) Heatmap of LC–MS/MS analysis. The intersection of high expressed proteins (Ratio > 3) and low expressed (bottom 100) in SW‐CM and PA‐CM in comparison with MIA‐CM. (B) Total differential proteins between SW‐CM and MIA‐CM. (C) Total differential proteins between PA‐CM and MIA‐CM. (D, E) GO and KEGG analysis of differential proteins

Proteomics analysis was performed to identify the proteins secreted by SW1990 and PANC‐1 cells (but not MIApaca‐2) that contributed to iCAF formation. The results indicated that proteins highly expressed in SW1990 and PANC‐1 cells showed an aggregation compared with MIApaca‐2 (Figure 4A). There were 878 proteins and 599 proteins that were upregulated in CM from SW1990 and PANC‐1, respectively, compared with that from MIApaca‐2 (Figure 4B and C). GO analysis showed that the upregulated proteins in PANC‐1 CM and SW1990 CM were mainly involved in cell secretion, extracellular matrix and fibroblast proliferation regulation (Figure 4D and E). This indicates that SW1990 and PANC‐1 may promote iCAF activation and tumour microenvironment formation through secretory proteins.

### 3.4 The effects of cancer cells secretory proteins on the prognosis of patients with PC

**FIGURE 5:**
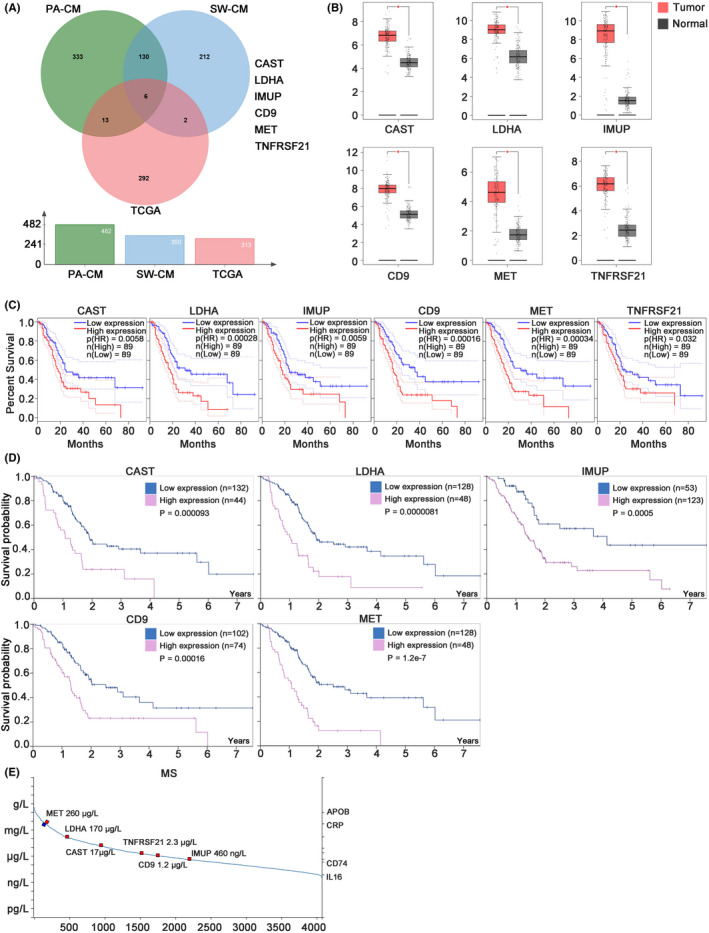
Six genes were selected and proved to be important roles in pancreatic cancer (PC). (A) The highly expressed proteins (ratio > 2) from PA‐CM and SW‐CM compared with MIA‐CM, and 313 PC survival‐related genes from the TCGA database (The genes were screened as *p* < 0.001) were used to screen candidate genes. (B) The mRNA level of six genes in tumour and normal tissues analysed in GEPIA. (C) The overall survival (OS) curve of six genes in PC by GEPIA. (D) The OS curve of six genes in PC by the Human Protein Atlas (HPA). (E) The protein level of six genes in human plasma was obtained from HPA.

A total of 313 PC survival‐related genes were obtained from the TCGA database (the genes were screened as *p* < 0.001). The intersecting genes between the upregulated proteins (ratio >2) from PANC‐1 and SW1990 CM and survival‐related genes from TCGA showed six genes—calpastatin (CAST), lactate dehydrogenase A (LDHA), immortalization up‐regulated protein (IMUP), CD9, tyrosine kinase receptor (MET) and tumour necrosis factor receptor superfamily member 21 (TNFRSF21) (Figure 5A)—which may play important roles in CAFs formation and PC tumour progression.

Bioinformatics analysis using GEPIA revealed that these six genes were all highly expressed in PC tumour tissues and were related to poor overall survival (OS) (Figure 5B and C).^19^ Among them, the expression of five genes (except TNFRSF21) correlated with disease‐free survival (Figure S2A). Meanwhile, bioinformatics analysis of data from UALCAN showed that the high expression of five genes (except CD9) was related to an advanced tumour grade (Figure S2B). Consistent with these results, data from The Human Protein Atlas verified that five genes (except TNFRSF21) had predictive significance for OS of PC patients (Figure 5D) (https://www.proteinatlas.org/). Moreover, according to the mass spectrometry data in The Human Protein Atlas, there were high levels of the six proteins in the plasma (Figure 5E). These results suggest that these six proteins may be valuable plasma prognostic markers for patients with PC.

### 3.5 The prognostic prediction values of six genes in PC

**FIGURE 6:**
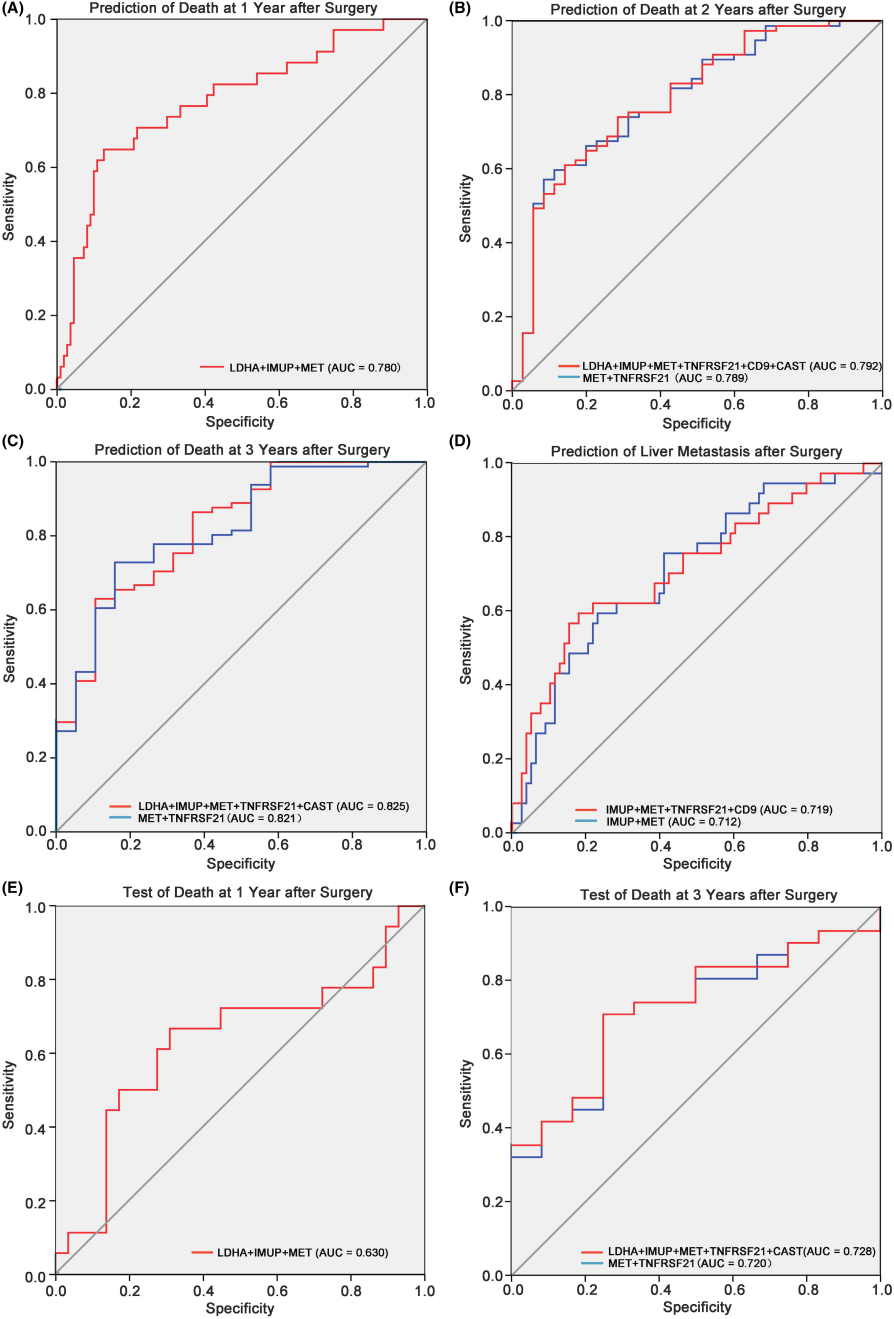
Predictive model of death risks and liver metastasis after surgery by six genes. (A–D) The ROC curve of prediction for 1‐ (A), 2‐ (B), 3‐year (C) death risk after surgery and liver metastasis risks (D) of the training model. (E, F) The ROC curve of prediction for 1‐ (E), and 3‐year (F) death risk after surgery of validation model

The ROC curve revealed that the multivariate model of the six genes and patient's age could predict the death risk of patients with PC after surgery. The AUC of LDHA, IMUP, MET and patient age score models performed on the prediction of death at 1 year after surgery was 0.780 (95% CI 0.686–0.874, *p* < 0.0001) (Figure 6A). In the prediction of death at 2 years after surgery, multivariate models of LDHA, IMUP, MET, TNFRSF21, CD9, CAST and age (AUC = 0.792, 95% CI 0.701–0.883, *p* < 0.0001) were superior to those of MET, TNFRSF21 and age (AUC = 0.789, 95% CI 0.698–0.880, *p* < 0.0001) (Figure 6B). Moreover, the model composed of LDHA, IMUP, MET, TNFRSF21, CAST and age had a prominent prediction of death at 3 years after surgery (AUC = 0.825, 95% CI 0.721–0.928, *p* < 0.0001) (Figure 6C). The prediction of liver metastasis after surgery using IMUP, MET, TNFRSF21, CD9 and age was also significant (AUC = 0.719, 95% CI 0.614–0.823, *p* < 0.0001) (Figure 6D). The clinical characteristics of the patients with complete clinical information in the different cohorts are shown in Table 1. Moreover, the 6‐gene expression data of tumour tissues from 48 patients with PC were detected. The logistic equation coefficients for 1‐, 2‐ and 3‐year mortality risk prediction are provided in Table S2. The externally validated AUC in the test of death at 1 year after surgery was 0.630 (95% CI 0.454–0.806, *p* = 0.137) (Figure 6 E). The test AUC of death at 3 years after surgery was 0.728 (95% CI 0.573–0.884, *p* = 0.021) for LDHA, IMUP, MET, TNFRSF21, CAST and age (Figure 6F). However, the external validation of death at 1 and 2 years after surgery were not statistically significant. This may be attributed to the small sample size of this study. Nevertheless, our data confirmed that these six genes have the potential to predict the prognosis of patients with PC after surgery with high sensitivity and specificity.

**TABLE 1:** Clinical characteristics of patients for training receiver operating characteristic analysis in different cohorts

	Death at 1 year after surgery (*n* = 139)			Death at 2 years after surgery (*n* = 106)			Death at 3 years after surgery (*n* = 95)			Liver metastasis after surgery (*n* = 109)		
Characteristics	Yes	No	*p*‐Value	Yes	No	*p*‐Value	Yes	No	*p*‐Value	Yes	No	*p*‐Value
Age (mean ± SD)	66.91 ± 11.11	64.23 ± 10.55	0.210^a^	66.82 ± 11.04	61.27 ± 9.72	0.018^a^ ^,*^	66.64 ± 10.99	60.80 ± 9.56	0.057^a^	62.19 ± 11.06	65.64 ± 10.68	0.120^a^
Gender												
Male (*n*, %)	20 (27.0)	54 (73.0)	0.425^b^	38 (70.4)	16 (29.6)	0.831^b^	39 (84.8)	7 (15.2)	0.554^b^	19 (32.2)	40 (67.8)	1.000^b^
Female (*n*, %)	13 (20.0)	52 (80.0)		38 (73.1)	14 (26.9)		41 (83.7)	8 (16.3)		17 (34.0)	33 (66.0)	
T classification												
T1 (*n*, %)	1 (25.0)	3 (75.0)	0.839^c^	1 (33.3)	2 (66.7)	0.047^c^ ^,*^	1 (100.0)	0 (0.00)	0.411^c^	1 (33.3)	2 (66.7)	0.719^c^
T2 (*n*, %)	4 (22.2)	14 (77.8)		8 (57.1)	6 (42.6)		9 (75.0)	3 (25.0)		3 (23.1)	10 (76.9)	
T3 (*n*, %)	28 (24.6)	86 (75.4)		66 (75.0)	22 (25.0)		69 (85.2)	12 (14.8)		32 (35.2)	59 (64.8)	
T4 (*n*, %)	0 (0.0)	3 (100.0)		1 (100.0)	0 (0.0)		1 (100.0)	0 (0.0)		0 (0.0)	2 (100.0)	
N classification												
N0 (*n*, %)	8 (19.5)	33 (80.5)	0.450^c^	15 (55.6)	12 (44.4)	0.032^c^ ^,*^	15 (71.4)	6 (28.6)	0.070^c^	9 (30.0)	21 (70.0)	0.680^c^
N1‐N4 (*n*, %)	25 (25.5)	73 74.5)		61 (77.2)	18 (22.8)		65 (87.8)	9 (12.2)		27 (34.2)	52 (65.8)	
AJCC Stage												
I (*n*, %)	4 (26.7)	11 (73.3)	0.303^c^	6 (46.2)	7 (53.8)	0.018^c^ ^,*^	6 (66.7)	3 (33.3)	0.100^c^	3 (27.3)	8 (72.7)	0.734^c^
II (*n*, %)	29 (24.8)	88 75.2)		67 (74.4)	23 (25.6)		71 (85.5)	12 (14.5)		33 (35.1)	61 (64.9)	
III (*n*, %)	0 (0.0)	3 (100.0)		1 (100.0)	0 (100.0)		1 (100.0)	0 (100.0)		0 (0.0)	2 (100.0)	
IV (*n*, %)	0 (0.0)	4 (100.0)		2 (100.0)	0 (100.0)		2 (100.0)	0 (100.0)		0 (0.0)	2 (100.0)	
Grade												
1 (*n*, %)	2 (10.0)	18 (90.0)	0.090^c^	8 (47.1)	9 (52.9)	0.025^c^ ^,*^	9 (60.0)	6 (40.0)	0.027^c^ ^,*^	4 (23.5)	13 (76.5)	0.010^c^ ^,*^
2 (*n*, %)	18 (23.4)	59 (76.6)		39 (73.6)	14 (26.4)		42 (87.5)	6 (12.5)		14 (24.6)	43 (75.4)	
3 (*n*, %)	13 (31.7)	28 (68.3)		28 (80.0)	7 (20.0)		28 (90.3)	3 (9.7)		17 (50.0)	17 (50.0)	
4 (*n*, %)	0 (0.0)	1 (100.0)		1 (100.0)	0 (0.0)		1 (100.0)	0 (0.0)		1 (50.0)	1 (50.0)	

Abbreviation: AJCC, American Joint Committee on Cancer.

^a^ Student's *t* test.

^b^ Chi‐Squared Test.

^c^ Mann–Whitney *U* test.

* *p* < 0.05.

## 4 DISCUSSION

Our study confirmed the crosstalk between PC cancer cell lines and PSCs. PSCs are activated by cancer cell secretory protein to form iCAF s and promote the migration and invasion of cancer cells. Previous studies have revealed the cancer‐promoting and cancer‐restraining roles of CAFs in PC, breast and prostate cancers.^8^, ^22^, ^23^ Tuveson et al.^9^ verified that IL‐1 and the transforming growth factor (TGF)‐β secreted by cancer cells could induce the formation of iCAF and myCAF, respectively. Another study uncovered the mechanism by which IL‐6/STAT3 signalling activation in hepatocytes leads to liver metastasis. Stromal cell CAFs release IL‐6, which is a trigger of liver metastasis signalling.^11^ Consistent with this, our study confirmed the upregulation of IL‐6, LIF and IL‐11 in iCAF after the activation of PSCs by CM from cancer cells. The proteins secreted by cancer cells greatly contribute to the formation of iCAF and PC progression.

In recent years, most researchers focused on the mechanisms by which CAF secretory proteins promote the invasion, metastasis and drug resistance in PC.^13^, ^14^, ^24^ However, the activation of CAFs from PSCs by cancer cells remains unclear. Some studies reported that tumour‐secreted IL‐1 and TGF‐β promoted stromal remodelling in PC.^9^, ^10^, ^12^ Vennin et al.^17^ identified that cancer cells with mutant p53 could induce invasive‐status CAFs to generate a pro‐metastatic and chemo‐resistant stromal microenvironment. Current pancreatic cancer cells, including SW1990, PANC‐1, BxPC‐3 and MIApaca‐2, all have p53 mutant status.^25^ Our results confirmed that CM from cancer cells, specifically SW1990 and PANC‐1 cells, promoted iCAF formation. We further demonstrated that the differentially expressed proteins secreted by SW1990 and PANC‐1 cells were mostly enriched in secretory regulation, fibroblast proliferation regulation and extracellular matrix formation. Among them, six genes were screened: CAST, CD9, IMUP, LDHA, MET and TNFRSF21. CAST, encoding an endogenous calpain inhibitor, plays an important role in c‐Myc mediated fibroblasts apoptosis.^26^ Moreover, CAST reduced the expression of TGF‐β and α‐SMA in burn‐wound fibroblasts and inhibited its proliferation.^27^ Notably, TGF‐β inhibits the activation of iCAF induced by tumour‐secreted IL‐1 and promotes the formation of myCAF.^9^ Therefore, CAST may promote the transformation of myCAF to iCAF. CD9 has been verified as a marker of PC stem cells and promotes tumour growth.^28^ IMUP was first identified in the immortalized fibroblasts and contributed to PC progression.^16^, ^29^ Thus, it may play a vital role in the immortalization of fibroblasts. In a study by Kang et al.,^30^ CAFs showed a higher level of glucose uptake, and the expression of LDHA was elevated in comparison with that in normal fibroblasts. Vigna et al.^31^ reported that MET inhibition reduced the activation of PSCs induced by PC cell lines. Moreover, they verified that the expression of fibroblast activation protein (FAP) and α‐SMA was reduced by MET inhibitor. Furthermore, fibroblasts do not express MET.^32^ Thus, MET should be secreted by the co‐cultured tumour cells. In addition, TNFRSF21 is reported to be involved in the transcription of inflammatory cytokines and fibrogenesis.^33^, ^34^ On the contrary, it is convenient to define the expression levels of the six genes since they can be detected in plasma. In summary, secretory proteins may play an important role in iCAF formation and malignant tumour stroma.

Due to the poor survival time after surgical resection, many studies have aimed to discover a prognostic marker to predict which patients with PC will benefit from surgical treatment.^35^, ^36^, ^37^ To date, there have been few biomarkers for therapeutic management in patients with PC, except for carbohydrate antigen 19–9.^38^ A recent study constructed a six‐gene model to predict PC metastasis. The AUC of the training and test groups was only 0.711 and 0.729, respectively.^35^ Another study established a prognostic risk score for the prediction of OS by five immune‐related genes. The external validation of the 1‐, 2‐ and 3‐year AUCs was 0.59, 0.75 and 0.77, respectively.^39^ In this study, we screened six genes that may lead to a poor prognosis by promoting iCAF activation and established a multiple‐gene model to predict the death risks at 1, 2 and 3 years and liver metastasis after surgery. In the training cohort of TCGA, the six genes had significant AUCs for predicting death at 1, 2 and 3 years after surgery and liver metastasis. However, due to the small number of clinical samples, the AUCs of death risks at 1 and 2 years after surgery and liver metastasis were not significant. The prediction of death at 3 years was externally validated with an AUC of 0.728 using LDHA, IMUP, MET, TNFRSF21, CAST and age, and 0.720 using MET, TNFRSF21 and age. These five‐gene models can be used as markers of poor response to surgical treatment for PC.

In conclusion, our study confirmed that cancer cells secreted proteins could promote iCAF formation, and that iCAF is a tumour‐promoting subtype in PC. Moreover, our data identified six alternative genes that may play a vital role in the activation of iCAF and tumour stromal formation. Thus, they may become therapeutic targets that target the stroma of PC. These six genes can also be used as predictive markers for death risk at 1, 2 and 3 years after surgical treatment. The preoperative plasma level of the 6‐gene model may suggest patients who could benefit from surgical resection in PC. However, the function and mechanism of these six genes need to be further explored and verified using large clinical plasma samples.

## AUTHOR CONTRIBUTIONS

**Qiankun Luo:** Writing – original draft (lead). **Jiayin Liu:** Software (equal); writing – original draft (equal). **Qiang Fu:** Data curation (equal); resources (equal). **Xu Zhang:** Investigation (equal); visualization (equal). **Pengfei Yu:** Validation (equal). **Pan Liu:** Data curation (equal). **Huiyuan Tian:** Data curation (lead); software (lead). **Hongwei Zhang:** Conceptualization (equal); formal analysis (equal); supervision (lead). **Song Chen:** Supervision (equal); writing – review and editing (lead). **Tao Qin:** Project administration (lead). **Jiali Zhang:** Writing – original draft (equal).

## FUNDING INFORMATION

This work was supported by the National Natural Science Foundation of China (31671440) and the Key Science and Technology Research Project of Henan Province (212102310151).

## CONFLICT OF INTEREST

The authors declare no competing financial interests.

## Supporting information


Figure S1

Figure S2

Tables S1–S2

Appendix S1

## Data Availability

All the data in this study are available from the corresponding author.
