# Supplementary figures and images for: Identifying cancer cell‐secreted proteins that activate cancer‐associated fibroblasts as prognostic factors for patients with pancreatic cancer
Source: J Cell Mol Med. 2022 Oct 25;26(22):5657–69. doi: 10.1111/jcmm.17596 (PMC9667520; doi:10.1111/jcmm.17596)

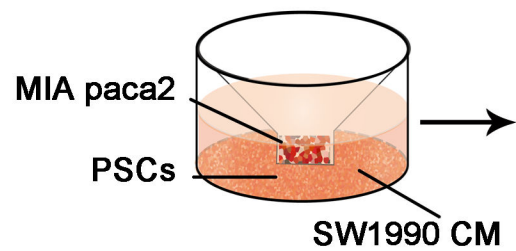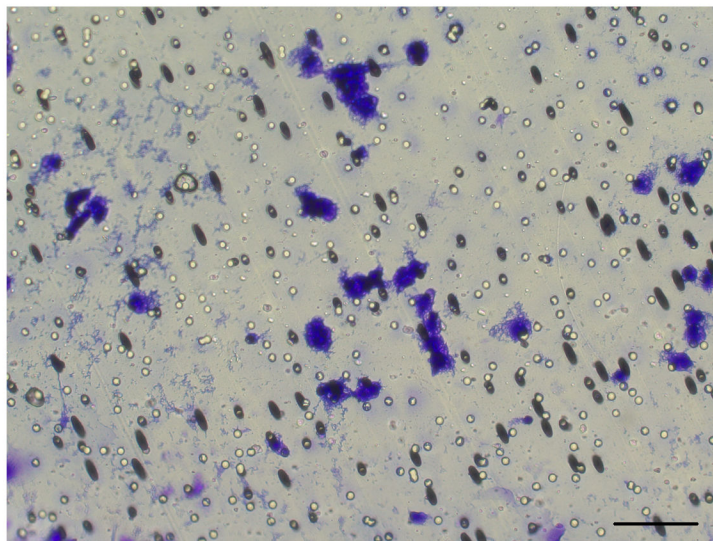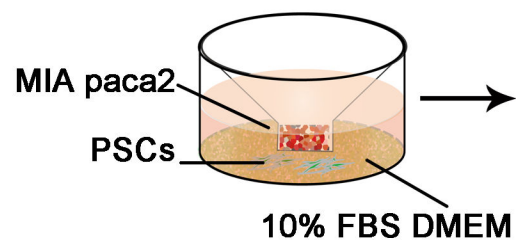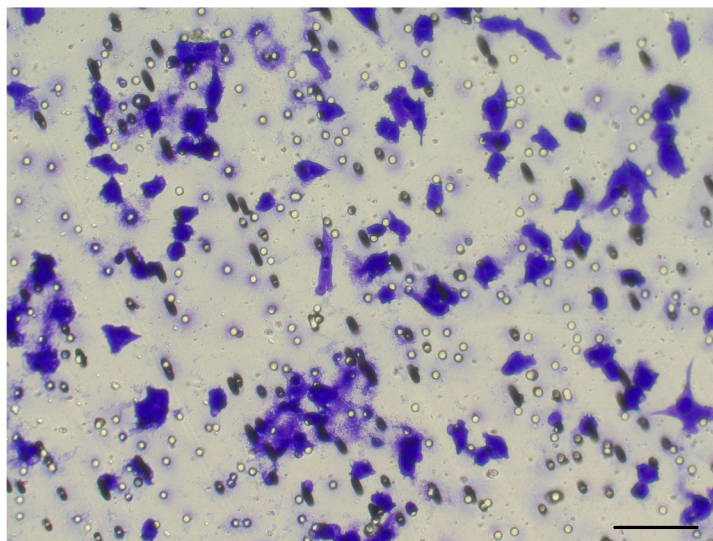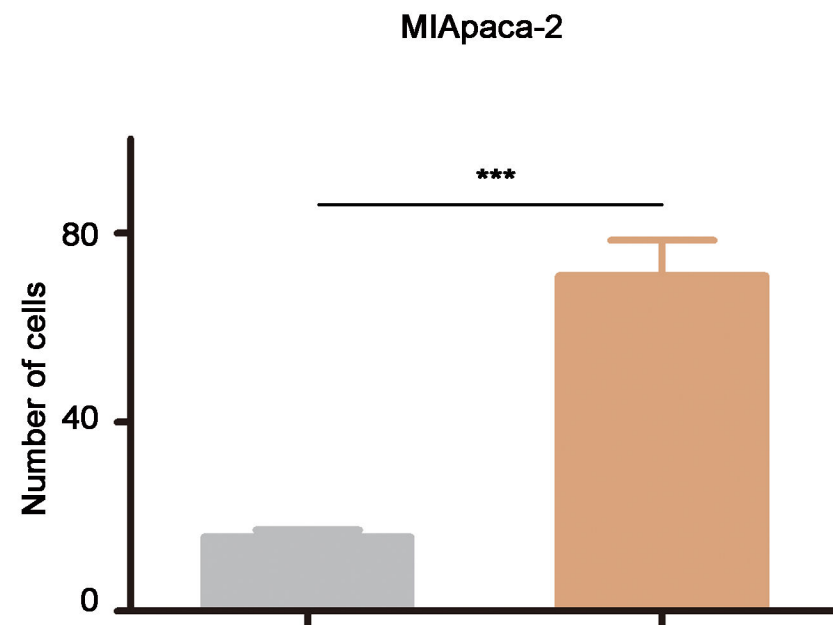

Supplement: Supplementary file 1 — Figure S1 [file JCMM-26-5657-s004.pdf]

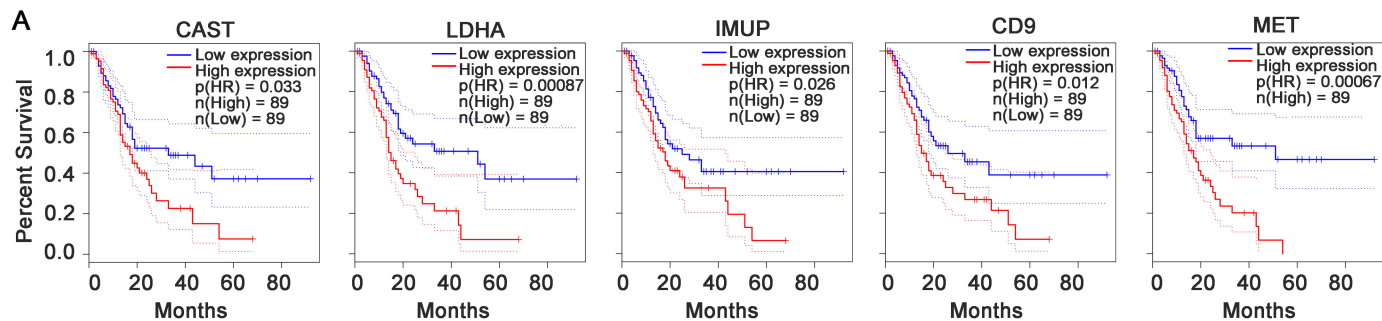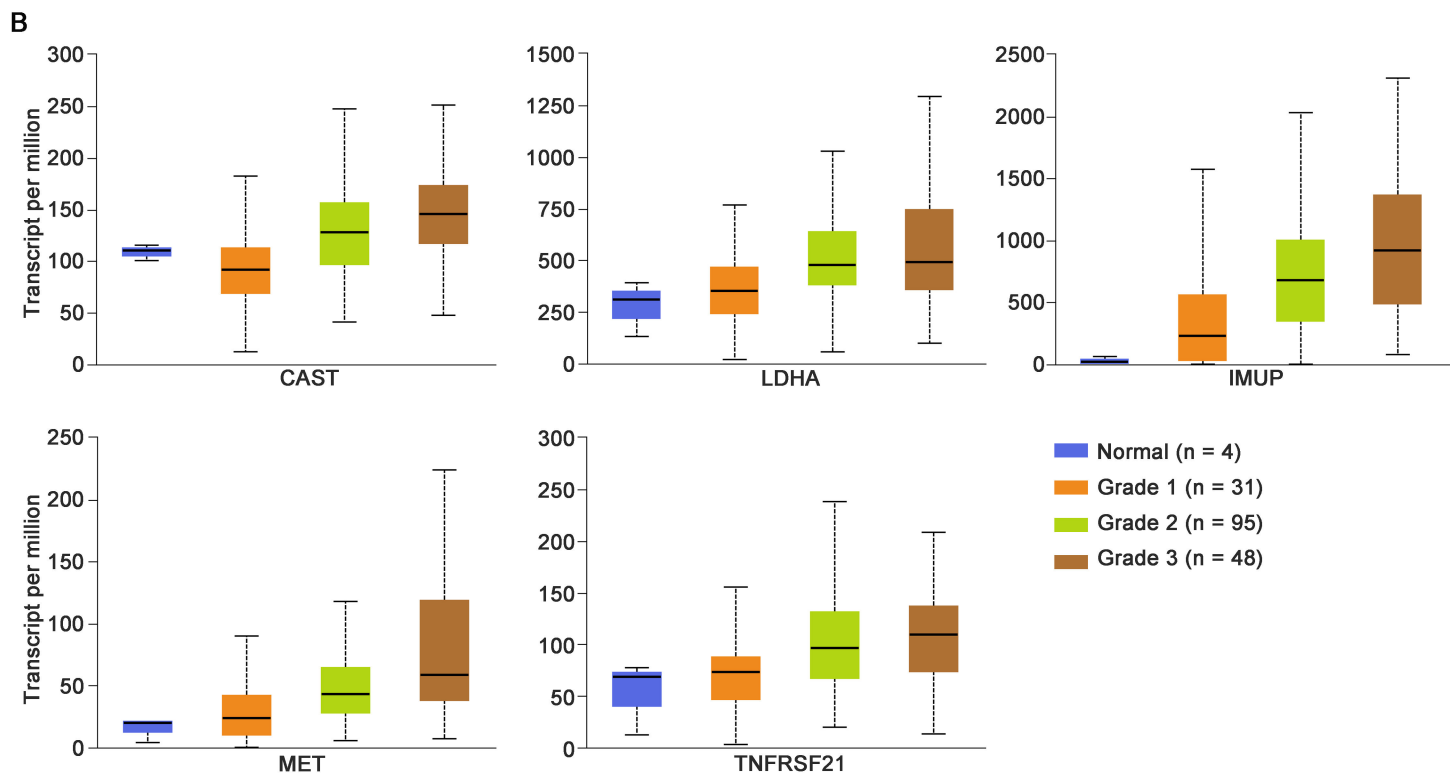

Supplement: Supplementary file 2 — Figure S2 [file JCMM-26-5657-s003.pdf]
