# Supplementary material for: Identifying cancer cell‐secreted proteins that activate cancer‐associated fibroblasts as prognostic factors for patients with pancreatic cancer
Source: J Cell Mol Med. 2022 Oct 25;26(22):5657–69. doi: 10.1111/jcmm.17596 (PMC9667520; doi:10.1111/jcmm.17596)
Supplement: Supplementary file 3 — Tables S1–S2 [file JCMM-26-5657-s002.pdf]

Supplementary Table S1. The primer sequences for RT-qPCR.

Gene	Forward 5'→3'	Reverse 5'→3'
CAST	TACAGGAAGTAACGATGCTCACA	CACCCGGCTTGCCAGATATT
LDHA	TTGACCTACGTGGCTTGGAAG	GGTAACGGAATCGGGCTGAAT
IMUP	GTCCGGGTCCAAAGCAAGG	GTGGGACTTCACATCCGTGT
CD9	CCTGCTGTTCGGATTTAACTTCA	TGGTCTGAGAGTCGAATCGGA
MET	AGCAATGGGGAGTGTAAAGAGG	CCCAGTCTTGTACTCAGCAAC
TNFRSF21	ATTGGCACATACCGCCATGTT	GGCTTGTGTTGGTACAATGCTC
β-actin	CCAACCGCGAGAAGATGA	CCAGAGGCGTACAGGGATAG

Supplementary Table S2. Logistic equation coefficient for 1-, 2-, and 3-year death risk prediction.

Death risk	Equation coefficient of logistic
1-year	age × 0.034 + IMUP × 0.001 + LDHA × 0.003 + MET × 0.007 - 5.504
2-year	* age × 0.064 + MET × 0.014 + LDHA × 0.001 + TNFRSF21 × 0.003 - CD9 × 0.001 - CAST × 0.003 - 4.849<br># age × 0.063 + MET × 0.015 + TNFRSF21 × 0.003 - 4.819
3-year	* age × 0.080 + MET × 0.032 + LDHA × 0.001 - TNFRSF21 × 0.001+ IMUP × 0.001 - CAST × 0.009 - 5.422<br># = age × 0.082 + MET × 0.032 + TNFRSF21 × 0.00 - 5.543

*, # representative logistic equation coefficient for 1-, 2-, and 3-year death risk prediction. The gene name refers to the gene expression quantity. IMUP, immortalization upregulated protein; LDHA, lactate dehydrogenase A; MET, tyrosine kinase receptor; TNFRSF21, tumor necrosis factor receptor superfamily member 21; CAST, calpastatin.
