# Supplementary material for: Identifying cancer cell‐secreted proteins that activate cancer‐associated fibroblasts as prognostic factors for patients with pancreatic cancer
Source: J Cell Mol Med. 2022 Oct 25;26(22):5657–69. doi: 10.1111/jcmm.17596 (PMC9667520; doi:10.1111/jcmm.17596)
Supplement: Supplementary file 4 — Appendix S1 [file JCMM-26-5657-s001.docx]

**Supplementary**

**Figure 1: Negative control of transwell co-culture.** MIApaca-2 were co-cultured in serum-free DMEM. In the bottom chamber, quiescent PSCs or SW1990 CM-induced PSCs were seeded in DMEM medium containing 10% FBS. The number of migration cells were analysed using Student's *t*-test. Bars, 50 μm.

**Figure 2: Expression of genes was related to disease-free survival (DFS) and tumour grade.** (A) Kaplan–Meier analysis of genes in DFS of PC patients by GEPIA. (B) The mRNA level of genes in different tumour grades of PC analysed by UALCAN.
